# Hyperbaric oxygen therapy outcomes in post-irradiated patient undergoing microvascular breast reconstruction: A preliminary retrospective comparative study

**DOI:** 10.1016/j.jpra.2024.07.017

**Published:** 2024-08-07

**Authors:** Matteo Scampa, Jérôme Martineau, Sylvain Boet, Rodrigue Pignel, Daniel F. Kalbermatten, Carlo M. Oranges

**Affiliations:** a Department of Plastic, Reconstructive, and Aesthetic Surgery, Geneva University Hospitals, Geneva University, 1205, Geneva, Switzerland; b Subaquatic and Hyperbaric Medicine Unit, Division of Emergency Medicine, Department of Anesthesiology, Clinical Pharmacology, Intensive Care and Emergency Medicine, Geneva University Hospitals and Faculty of Medicine, University of Geneva, 1205, Geneva, Switzerland; c Department of Anesthesiology and Pain Medicine, The Ottawa Hospital, Ottawa, ON, K1H 8L6, Canada; d Ottawa Hospital Research Institute, Clinical Epidemiology Program, Department of Innovation in Medical Education, University of Ottawa, Ottawa, ON, K1H 8L6, Canada; e Institut du Savoir Montfort, Ottawa, ON, K1K 0T2, Canada

**Keywords:** Hyberbaric, Oxygenotherapy, HBOT, Autologous, Breast, Flap

## Abstract

**Introduction:**

Radiotherapy is a challenge in autologous breast reconstruction because of its impact on cutaneous and vascular systems. Hyperbaric oxygen therapy (HBOT) is a recognized treatment of radiation-related complications. We aimed to assess the impact of perioperative HBOT on irradiated breast microvascular reconstructive outcomes.

**Method:**

We reviewed the medical charts of patients who received radiotherapy and then underwent secondary free autologous breast reconstruction at our institution. Data on demographics, HBOT protocol, intervention characteristics and post-operative complications were collected. Outcomes of the irradiated patients were then compared between the HBOT and non-HBOT groups.

**Results:**

Fourteen patients were included (11 unilateral and 2 bilateral deep inferior epigastric artery perforator flaps and 1 free transverse rectus abdominis muscle flap). Seven patients received HBOT and 7 did not. In the non-HBOT group, there were 1 Clavien–Dindo grade II, 1 Clavien–Dindo grade IIIa and 2 Clavien–Dindo grade IIIb post-operative complications. In the HBOT group, there were 3 Clavien–Dindo grade I, 1 Clavien–Dindo grade IIIa and 2 Clavien–Dindo grade IIIb post-operative complications. The mean operative time was 452.3 minutes (SD ±62.4 minutes) for unilateral cases without HBOT and 457.8 minutes (SD ±102.1 minutes) with HBOT (*p*=0.913). Mean ischaemia time per flap without HBOT was 109.4 minutes (SD ±51.8 minutes) versus 80.1 minutes (SD ±37.7 minutes) in the HBOT group (*p*=0.249).

**Conclusion:**

This study provides insights into the potential of HBOT treatment in preparing patients with irradiated breast cancer for secondary autologous reconstruction.

## Introduction

Breast cancer treatment mainly revolves on the surgical excision of the mammary gland, often preceded or followed by systemic treatment or radiotherapy.^1^ These surgeries induce considerable alterations in the patient's body. Although not mandatory, breast reconstruction is part of the treatment and allows to improve the patient's well-being.^2^ Breast reconstruction after mastectomy primarily relies on implant-based breast reconstruction or autologous reconstruction. Radiotherapy is frequently used as an adjuvant therapy after conservative surgery (tumorectomy) or following mastectomy in patient with advanced local disease or regional invasion.^3^ However, radiation therapy induces fibrosis of the targeted area and surrounding tissues, which can jeopardize the breast reconstruction outcomes.^4^ According to the literature, patients who undergo immediate implant-based breast reconstruction and receive radiotherapy have an estimated risk of 16.9% to 67.5% to develop capsular contracture.^5^^,^^6^ In our practice, secondary autologous breast reconstruction is generally offered to patients who develop radiation-related complications after implant-based reconstruction and also to patient who wish to undergo implant removal. Autologous breast reconstruction has been demonstrated to be associated with better patient-reported satisfaction rates than implant-based reconstruction.^7^ Despite bringing healthy tissues to the recipient site, adverse surgical events remain frequent after such complex microsurgical procedures and require new strategies to optimize surgical outcomes.^8^ Hyperbaric oxygen therapy (HBOT) has been shown to improve skin quality after cutaneous radiation injury by promoting neo-angiogenesis in irradiated tissues. HBOT involves the administration of 100% FiO_2_ to patients at an atmospheric pressure of approximately 2 atm in consecutive sessions of variable length. This procedure acts by facilitating oxygen diffusion in tissues. In case of an ischaemic insult or ischaemia-reperfusion injury, this mechanism allows better oxygenation of the compromised tissues.^9^, ^10^, ^11^ It further acts by providing an alternation between hyperoxygenation phases (during HBOT sessions) and hypoxic phases (in between HBOT sessions), which stimulates fibroblasts, allowing angiogenesis and increased collagen deposition.^12^ A similar effect on tissues affected by radiation damage has been demonstrated.^13^ It is an established technique used in the perioperative setting to improve radiation-related complications, but evidence on its efficacy remains limited.^14^^,^^15^ Although the safety of HBOT in an oncological setting has been previously questioned, current evidence suggests that it is a safe procedure and it can also have an inhibitory effect on the growth of cancer cells.^16^ This preliminary study aimed at assessing whether perioperative HBOT translates into improvements in surgical outcomes during autologous breast reconstruction in previously irradiated patients. The role of HBOT in improving flap salvage in case of vascular compromise has been widely reported and prophylactic HBOT has been shown to improve surgical outcomes.^9^^,^^17^^,^^18^ HBOT utilization has also been demonstrated to be successful in treating post-operative complications after breast surgery.^19^ However, there is no evidence on the role of prophylactic HBOT in preventing post-operative complications in the specific context of post-radiation breast surgery. We hypothesized an improvement in surgical outcomes with pre- and post-operative HBOT in the setting of autologous breast reconstruction in irradiated patients with breast cancer.

## Method

STROBE guidelines were followed for manuscript elaboration.

After obtaining institutional review board approval (CCER 2024-00007), a retrospective chart review of all consecutive female patients with breast cancer who underwent secondary microsurgical autologous reconstruction (free flap) between October 2020 and January 2024 at the plastic surgery department of Geneva University Hospital by a single surgeon (CMO) was conducted. Inclusion criteria included the use of adjuvant radiotherapy on the operated breast before autologous reconstruction. Patients who did not provide a signed general consent for research were excluded from the study. Because operative and early post-operative outcomes were studied, a minimal follow-up period of 30 days was required. The patient's demographic information, oncologic characteristics, surgical details, HBOT characteristics and follow-up data were extracted in March 2024 from the patient's medical files (operative and consultation reports) and analysed. Most patients undergoing autologous breast reconstruction after radiation therapy were offered HBOT at the senior author's discretion. However, not all patients accepted and, in some cases, the required logistical setting could not be provided. All instances for which HBOT was used, that is, post-mastectomy HBOT, pre-autologous reconstruction HBOT and post-autologous reconstruction HBOT were recorded. The primary outcome was post-operative complications classified according to Clavien–Dindo system.^20^ All complications that occurred during the hospital stay and outpatient follow-up were recorded, and the highest complication grade was reported. For bilateral cases, complications were counted on a per patient basis. We further analysed the impact of pre-operative HBOT on intraoperative outcomes (ischaemia and operative times). Patients who did not receive prophylactic pre-operative HBOT were not included for the intraoperative outcomes (ischaemia and operative times). Bilateral cases were not included in operative time statistics to avoid bias owing to their expected longer operative time. Ischaemia time was computed on a per breast basis for bilateral cases because flap weaning and anastomosis were performed consecutively on each side, allowing the recording of ischaemia time for each breast in bilateral patients. Patients’ complications were then compared between those who received HBOT and those who did not. Operative and ischaemia times were compared between HBOT and non-HBOT patients using the student's *T*-test after testing the equality of variances assumption using Levene's test. The JASP V.0.18.3 software was used for statistical analysis.^21^ A p-value <0.05 was considered significant.

## Results

Fourteen consecutive patients met the selection criteria (Table 1). No patients were excluded. The mean population age was 56 years (SD ±11 years). Mean body mass index was 26.7 kg/m^2^ (SD ±3.3 kg/m^2^). All reconstruction were secondary with 11 unilateral deep inferior epigastric artery perforator (DIEP), 2 bilateral DIEP and 1 free transverse rectus abdominis muscle (free-TRAM). Indications for autologous reconstruction were periprosthetic capsulitis in 9 cases, 1 patient who did not initially have implant-based reconstruction owing to compromised mastectomy flap, 1 direct closure after mastectomy (secondary autologous reconstruction was previously decided with the patient), 1 patient with implant infection who required implant removal, 1 patient with post-operative skin necrosis associated with implant exposure and 1 autologous breast reconstruction wished by the patient. Seven patients did not receive HBOT. In the 7 other cases, HBOT was used, but with variable timing (Table 1). Six cases received prophylactic pre-operative HBOT. Case 12 only had post-operative HBOT and was therefore not included in the secondary outcomes analysis (ischaemia and operative times). All patients received standard HBOT sessions consisting of 100% oxygen inhalation at 2.5 atm for 95 minutes (including pressurisation and depressurisation), except for case 11 who also had 1 session in between standard sessions during which the pressure was lower at 2.0 atm.

**Table 1:** Population characteristics.

Case	Age (year)	Flap type	Side	HBOT	Other oncologic treatments	Operation length (min)	Ischaemia time (min)	Flap weight (g)	Length of stay post-op (day)	Reason for autologous breast reconstruction
1	63	f-TRAM	R	No	Adj Hx	420	80	N/R	8	Periprosthetic capsulitis
2	42	DIEP	L	No	Neo Cx, Ix; Adj Ix, Hx	570	146	275	12	Periprosthetic capsulitis
3	61	DIEP	L	No	Neo Hx	400	170	340	8	Periprosthetic capsulitis
4	72	DIEP	R	No	Adj: Hx	480	173	624	8	Periprosthetic capsulitis
5	65	DIEP	L	No	No	420	68	N/R	16	Periprosthetic capsulitis
6	44	DIEP	L	No	Adj: Hx	480	79	N/R	7	Periprosthetic capsulitis
7	37	DIEP	R	No	Neo: Cx	396	50	550	9	No reconstruction after mastectomy
8	59	DIEP	R	Yes 11 pre-op	Adj: Hx	451	N/R	N/R	8	Post-operative skin necrosis with implant exposure
9	43	DIEP	B	Yes 11 pre-op 5 post-op	Neo: Cx	800	Left 75 / Right 115	N/R	9	Implant infection
10	59	DIEP	B	Yes 15 pre-op 52 post-op	Neo: Cx Adj: Ix	489	Left 35 / Right 66	N/R	23	Periprosthetic capsulitis
11	73	DIEP	R	Yes 16 pre-op	Neo: Cx Adj: Hx	420	66	N/R	9	Periprosthetic capsulitis
12	57	DIEP	L	Yes 29 after initial mastectomy 9 post-op	Adj: Hx	540	101	N/R	9	Patient's wish
13	54	DIEP	R	35 after initial mastectomy 18 pre-op	Adj: Cx, Ix, Hx	360	58	N/R	8	No reconstruction after mastectomy owing to compromised mastectomy flap
14	57	DIEP	R	Yes 8 pre-op 30 post-op	Adj: Hx	600	146	N/R	11	Periprosthetic capsulitis

R: Right; L: Left; B: Bilateral; Adj: Adjuvant; Neo: Neo-adjuvant; Cx: Chemotherapy; Hx: Hormonotherapy; Ix: Immunotherapy; N/R: not reported

In the non-HBOT group, there were 1 Clavien–Dindo grade II, 1 Clavien–Dindo grade IIIa and 2 Clavien–Dindo grade IIIb post-operative complications. In the HBOT group, there were 3 Clavien–Dindo grade I, 1 Clavien–Dindo grade IIIa and 2 Clavien–Dindo grade IIIb post-operative complications. (Table 2).

**Table 2:** Post-operative complications.

Cases	Clavien–Dindo classification	Complications
1	0	
2	0	
3	II	Urinary tract infection treated with antibiotics and nipple partial necrosis (conservative treatment)
4	IIIa	Nipple partial necrosis and recurrent reconstructed breast seroma (US-guided aspiration)
5	IIIb	Donor-site haematoma surgically evacuated with subsequent surinfection
6	IIIb	Abdominal wound dehiscence requiring surgical revision
7	0	
8	IIIa	Breast and abdomen wound dehiscence requiring revision under local anaesthesia
9	I	Wound dehiscence (conservative treatment)
10	IIIb	Abdominal skin necrosis requiring a distant skin graft and blood transfusion
11	0	
12	IIIb	Breast haematoma requiring surgical revision, breast wound dehiscence requiring revision under local anaesthesia, abdomen wound dehiscence (conservative treatment) and blood transfusion
13	I	Breast wound dehiscence (conservative treatment)
14	I	Pleural effusion (diuretic treatment + CPAP)

**Figure 1:**
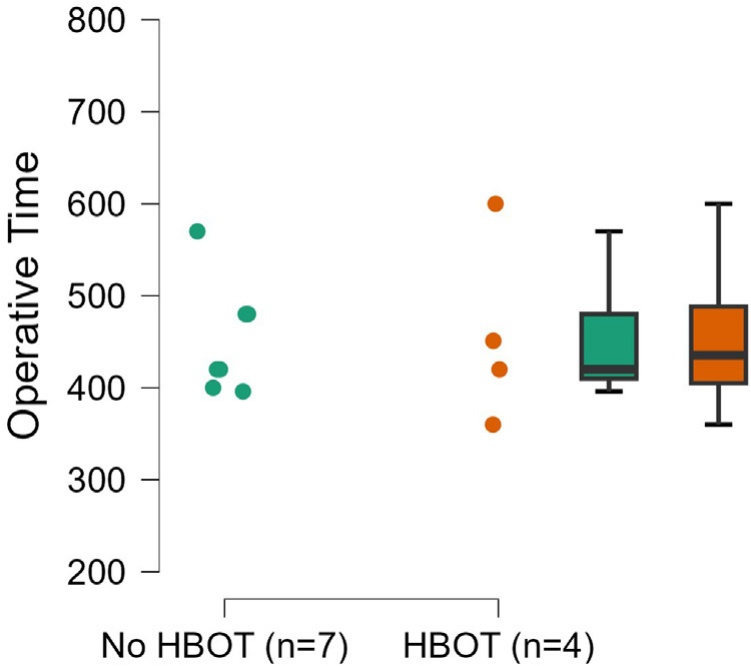
Dot plot of operative time.

**Figure 2:**
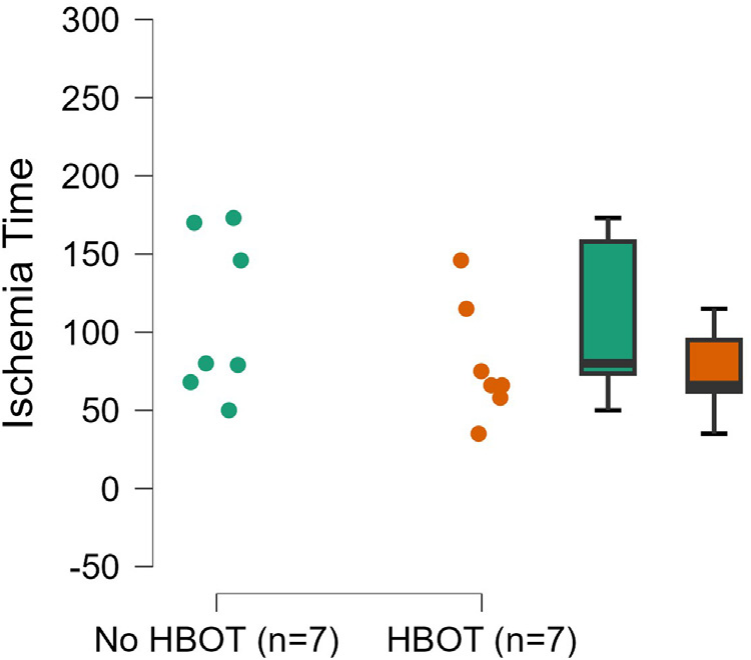
Dot plot of ischaemia time.

The overall mean operative time including bilateral cases (n=13) was 483.5 minutes (SD ±117 minutes). Mean operative time in unilateral cases (n=7) without HBOT was 452.3 minutes (SD ±62.4 minutes) compared with 457.8 minutes (SD ±102.1 minutes) in unilateral cases with pre-operative HBOT (n=4). No significant difference in operative time was observed between unilateral patients with HBOT and those without (p=0.913) (Figure 1). Ischaemia time was reported in 12 cases, one patient had missing data in his medical chart and case 12 was excluded. The overall mean ischaemia time (n=14 breasts) was 94.8 minutes (SD ±46.1 minutes). Mean ischaemia time per breast in patients without pre-operative HBOT (n=7 breasts) was 109.4 minutes (SD ±51.8 minutes), versus 80.1 minutes (SD ±37.7 minutes) in the HBOT group (n=7 breasts) (p=0.249) (Figure 2). Mean hospitalisation duration after the reconstruction was 9.7 days (SD ±3.0 days) in the non-HBOT group and 11 days (SD ±5.0 days) in the HBOT group (p=0.648). Flap weight was reported only in 4 cases (patients 2, 3, 4 and 7).

The mean follow-up duration was of 325.7 days (SD ±201.1 days). During the follow-up period, 1 patient died from breast cancer during the study period.

## Discussion

In this preliminary study, we describe the prophylactic use of pre-operative HBOT for autologous microsurgical breast reconstruction in patients who previously underwent radiotherapy. However, no statistically significant results were found. Interpretation of the results remains limited by the retrospective design of the study owing to incomplete data and potential intervention bias. Although HBOT was offered to most patients, the therapy selection was at the discretion of the senior author, highlighting a potential intervention bias in patients who are more likely to develop post-operative complications owing to comorbidities or stronger radiation sequalae. A prospective randomized study is required to confirm a potential benefit from pre-operative HBOT to optimize surgical outcomes in previously irradiated patients.

The selected outcomes are representative of the overall surgical outcomes. Although post-operative complications are directly related to surgical success, operation length and ischaemia time indirectly translate the potential difficulties encountered during the reconstruction and microsurgical anastomosis. We also described complications that occurred after the initial hospitalisation, with a mean of 325 days of follow-up, ensuring an exhaustive assessment of late post-operative complications that may occur after such procedures.

The study population was exclusively composed of patients undergoing secondary autologous reconstruction, because we believe that the combination of mastectomy with autologous reconstruction may result in longer operative times and pose higher anaesthesia-related risks for our patients. Furthermore, if radiation is required after definitive pathology results, we aimed to prevent skin damage of the reconstructed flap. Whenever feasible, immediate pre-pectoral silicone implant-based reconstruction was offered to all our patients and autologous reconstruction was subsequently proposed, either upon the patient's request or in instances of capsular contracture, after the completion radiation therapy. In our case series, only 1 patient (case 8) required autologous reconstruction a few days after mastectomy as she developed an extended mastectomy flap necrosis with implant exposure. She had a history of prior breast conservative surgery for breast cancer with adjuvant radiotherapy. Furthermore, mastectomy was performed in the context of a relapse 9 years after the initial cancer. Despite post-mastectomy HBOT, the mastectomy flap skin could not be saved and we offered the patient autologous breast reconstruction for salvage. In this case, the post-mastectomy HBOT was considered as prophylactic pre-autologous breast reconstruction HBOT.

We believe that HBOT may facilitate flap in-setting by improving vascularity, cellularity and collagen deposition around the thoracic wound edges. In our experience, recipient vessels for breast reconstruction are often affected by brittle walls, intimal dissection and higher susceptibility to breakage during manipulation after radiotherapy. Moreover, higher rates of intimal dissociation, artery hyalinosis and decreased media thickness have been described in literature.^22^, ^23^, ^24^ A histological study on rats demonstrated diminution of endothelial cells, of nuclei in the smooth muscle cells, of the media with oedema and fibrosis, of the adventitia in veins and of arteries after radiotherapy, but only found higher thrombosis rates in veins.^25^ Furthermore, previous chemotherapy associated with radiotherapy also influences the quality of the recipient vessels.^26^ In the present case series, 6 out of 14 patients had concomitant radiotherapy and chemotherapy (2 in the non-HBOT and 4 in the HBOT groups), also potentially explaining longer operative time and ischaemia time in these cases. Chemotherapy was administered as a neo-adjuvant treatment in 5 patients, whereas 1 patient received adjuvant chemotherapy with >1 month between the last injection and autologous breast reconstruction. The small size of the cohort did not allow for the analysis of the influence of chemotherapy and post-operative complications, surgical or ischaemia time. However, neo-adjuvant or adjuvant chemotherapy is not associated with increased complications after immediate breast reconstruction according to a strong multicentric study.^27^

Literature demonstrated higher failure rates when free flap reconstruction was attempted on irradiated vessels.^28^ With pre-operative HBOT, we attempted to improve microsurgical outcomes by enhancing the quality of the recipient vessel. However, no details of the vessel quality and necessity to redo the anastomosis was reported in the medical records, therefore their analysis was not performed. However, no vascular compromise was reported in either group. When analysing ischaemia time, we found shorter times in the HBOT group, albeit not statistically significant. Interestingly, in the 2 patients with bilateral DIEP, we found shorter ischaemia time in the irradiated breast compared with the contralateral one. However, this trend was not reflected in the average operating time, with longer operations in the HBOT group.

The number of HBOT sessions varied slightly among patients, with some having undergone prior HBOT at the time of mastectomy due to mastectomy skin flap compromise. We aimed to adhere to a protocol of 20 pre-operative sessions followed by 10 post-operative sessions. However, logistical constraints and patients’ restrictions prevented strict adherence to this protocol. Regarding the post-operative sessions, the number of sessions could be further increased in the event of complications. Overall, HBOT was well tolerated by our patients with good compliance, even in the ambulatory setting with no reported HBOT-related complication.

When assessing post-operative complications, only one immediate complication of the recipient site (breast) required an emergency surgical hematoma evacuation, the same patient later developed wound dehiscence that required distant revision under local anaesthesia for correction (case 12). This patient had received HBOT following the initial mastectomy but did not receive the HBOT regimen before the autologous reconstruction. Other complications were on the donor site (not affected by radiotherapy) and conservatively treated. Case 5 presented a donor-site hematoma requiring operative takeback during the hospital stay, which was further complicated by a donor-site infection by *Pseudomonas aeruginosa*. Case 10 experienced extensive necrosis of the abdominoplasty flap despite prolonged post-operative HBOT, requiring iterative ambulatory debridement with negative pressure wound therapy, followed by a split-thickness skin graft reconstruction. The most frequent complication described was wound dehiscence or delayed wound healing that were mostly managed conservatively or with surgical treatment under local anaesthesia. They are equally represented in the non-HBOT (n=4) and HBOT (n=5) groups. Patients in the HBOT group appeared to have more wound healing problems in the breast than those in the non-HBOT group. This could potentially be explained by an intervention bias where patients with more severe cutaneous radiation injury may be more readily offered HBOT. A prospective randomized study is required to investigate the effects of HBOT on wound healing problems and draw conclusions.

The length of hospital stay appeared to be longer in the HBOT group, despite no statistical significance. It can be explained by the presence of 2 bilateral reconstructions in the HBOT group and none in the non-HBOT group. Usually, bilateral reconstruction is associated with longer operative times, which is correlated with higher complications rates in unilateral and bilateral autologous breast reconstruction.^29^^,^^30^

## Conclusion

This study provides insights into the potential of HBOT to prepare irradiated patients with breast cancer for secondary autologous reconstruction. However, the retrospective study design limits the ability to draw conclusions owing to potential intervention bias. A prospective randomized intervention study is required to confirm whether ischaemia time, operative time and complication rates can be reduced by using prophylactic pre-operative HBOT.
